# Supplementary material for: Enhanced neural plasticity in monkey TE compared to TEO during learning of a feature-ambiguous visual categorization task
Source: Nat Commun. 2025 Dec 10;16:11374. doi: 10.1038/s41467-025-66387-3 (PMC12727691; doi:10.1038/s41467-025-66387-3)
Supplement: Supplementary file 1 — Supplementary Information [file 41467_2025_66387_MOESM1_ESM.pdf]

Supplementary Information

Enhanced neural plasticity in monkey TE compared to TEO during learning of a feature-ambiguous visual categorization task

Wenliang Wang[1, †], Bing Li[1, †], Narihisa Matsumoto[2, †], Kariely Martinez Gomez[1], Kazuko Hayashi[2], Yasuko Sugase-Miyamoto[2], Richard C. Saunders[1], Barry J. Richmond[1]*, Mark A. G. Eldridge[1,3]*

[1]Laboratory of Neuropsychology, National Institute of Mental Health, National Institutes of Health, Bethesda, MD, USA

[2]Human Informatics and Interaction Research Institute, National Institute of Advanced Industrial Science and Technology (AIST), Tsukuba, Ibaraki, Japan

[3]Present address: Biosciences Institute, Newcastle University, Newcastle upon Tyne NE1 7RU, UK

[†]These authors contributed equally

*Correspondence: mark.eldridge@newcastle.ac.uk; barryrichmond@mail.nih.gov

Content of this file:

Supplementary Fig. 1 – 12

Supplementary Notes

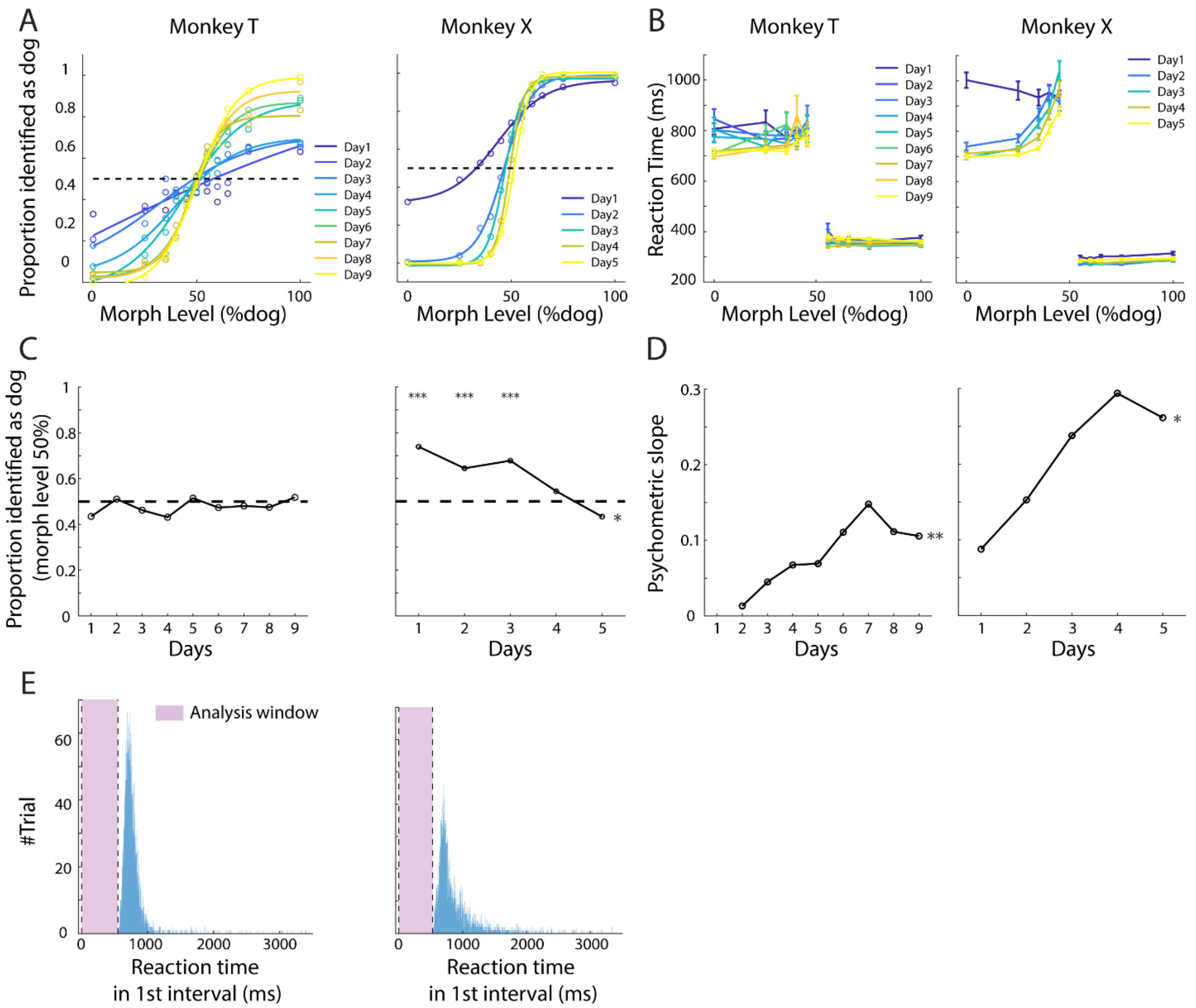

Supplementary Fig. 1. Categorization performance of monkeys during learning. (A) Percentage identified as dog for monkey T and X across learning days. Data fit with the logistic function: a + b / (1 + exp (- c * (x - d))). Fitted curves with an adjusted R^2 below 0.85 (i.e., monkey T's first day) were not displayed. (**B**) Reaction times. Reaction times for correct bar releases in the first interval for cat-like images (left) decreased significantly across learning days. T: $p = 0.006$, $F_{(8, 402)} = 2.72$; X: $p = 3.17 \times 10^{-25}$, $F_{(4, 468)} = 34.46$, two-way ANOVA. Reaction times for correct bar releases in the second interval for dog-like images were defined as the time between the onset of the green square and the moment the bar was released. (**C**) Categorization performance of monkeys on 50% morphed images. Choice bias was evaluated by monkeys' choices on 50% morphed images. For monkey T, no significant biases were observed in all the days. $p > 0.05$ for all days, chi-square test; For monkey X, significant biased choices were observed in the first 3 days. $p = 2.37 \times 10^{-10}$, 1.4×10^{-4}, 2.66×10^{-6}, 0.26, 0.09 (day 1-5); $\chi^2_{(1)}$ = 40.14, 14.45, 22.05, 1.25, 2.94, indicated by asterisks on the top, chi-square test. Choice biases were improved across days for monkey X. $p = 0.02$, $r = -0.93$, Pearson correlation, indicated by the asterisk on the side. (**D**) The slope of the psychometric curve ('c' in the above function)

increased across learning days for both monkeys. $p = 0.007$, 0.03, $r = 0.85$, 0.91, for T and X, respectively, Pearson correlation. (**E**) Distribution of reaction times when monkeys release the bar in the first interval (on red dot). Shaded areas indicated time windows when neural responses were analyzed in the present study. Data are mean ± SEM; $*p < 0.05$, $**p < 0.01$, $***p < 0.001$.

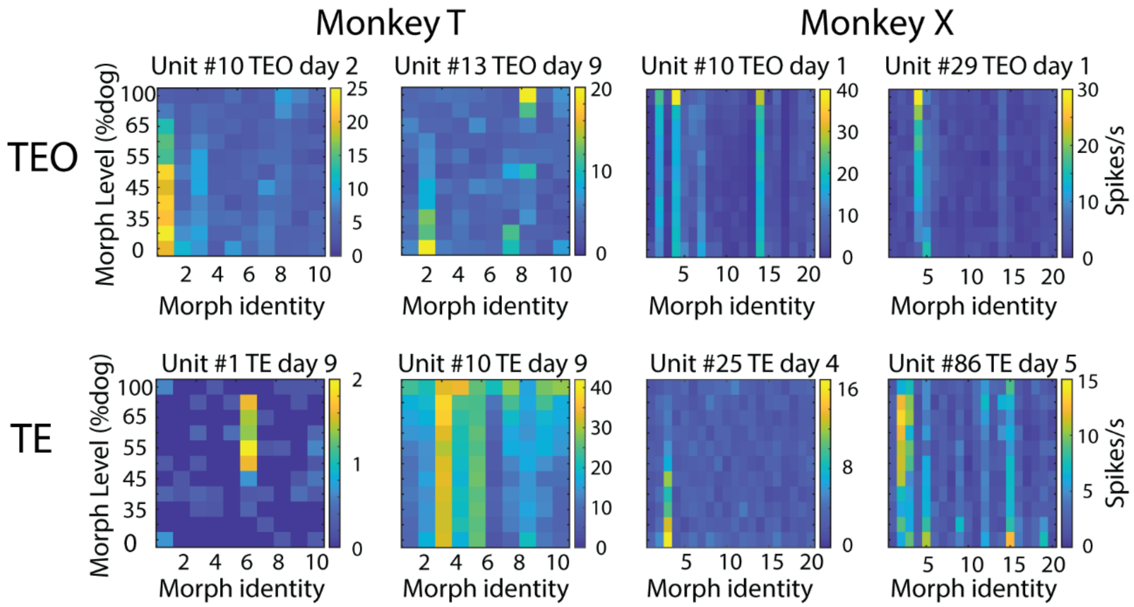

Supplementary Fig. 2. Responses of example neurons from TE and TEO. Each panel is a color-coded response matrix showing a single neuron's mean firing rate (spikes s^{-1}) to morphed cat–dog images. Warmer colors indicate higher firing. Color bars on the right show panel-specific scaling. Panels are grouped by animals (left: Monkey T; right: Monkey X) and brain regions (top row: TEO; bottom row: TE). Titles list unit ID and recording day. The x-axis indexes the morph identities (10 for Monkey T; 20 for Monkey X). The y-axis (Morph level, % dog) runs from 0% (i.e., 100% cat) at the bottom to 100% dog at the top.

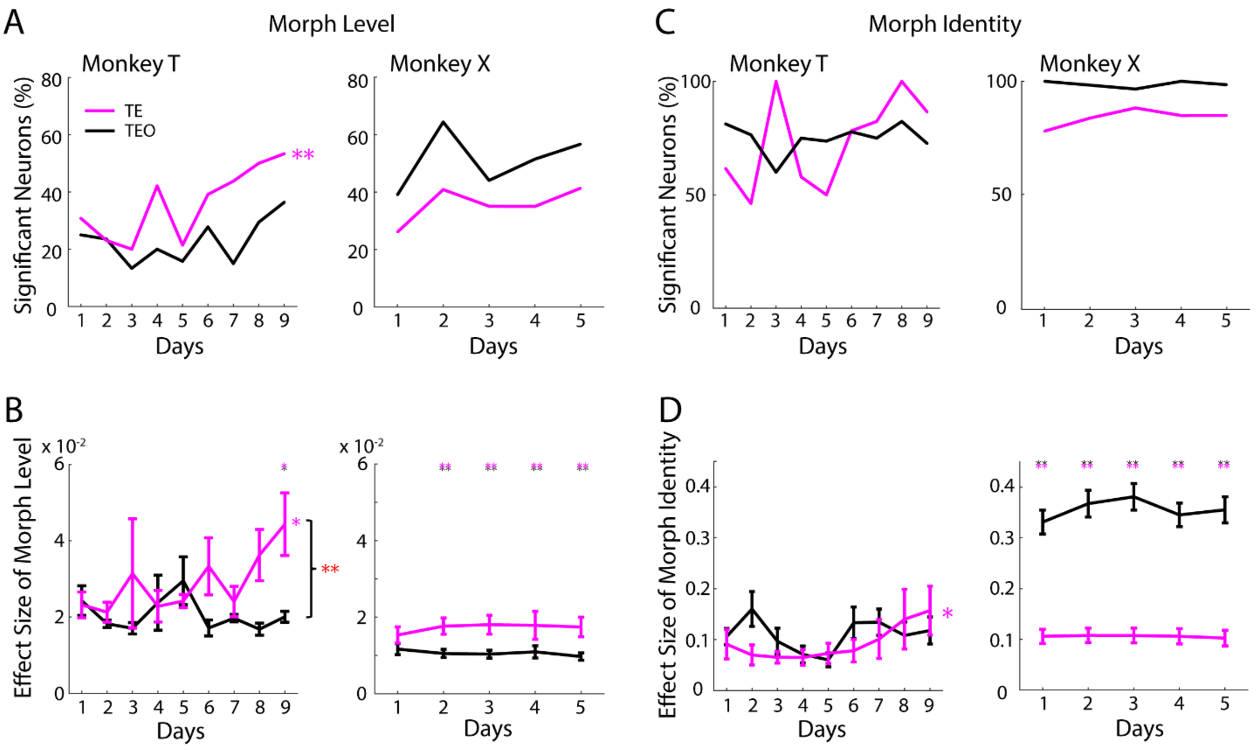

Supplementary Fig. 3. Effects of morph level and morph identity on neuronal responses in TE and TEO. (**A**) Percentage of neurons significantly modulated by morph level in TE (magenta) and TEO (black) during learning. T: $p_{(\text{percentage-vs-day})} = 0.13$ (TEO), 0.0029 (TE), r = 0.55, 0.86; X: p = 0.52, 0.35, r = 0.4, 0.6, Pearson correlation. Lower bounds of one-tailed 95% confidence interval (CI) of $r_{(\text{TE-vs-day})} - r_{(\text{TEO-vs-day})}$: -0.16 (T), -0.63 (X), bootstrapping Pearson correlation (5000 bootstrap samples). (**B**) Effect size of morph level on TEO and TE neurons which were significantly modulated by morph level. T: $p_{(\text{effect-size-vs-day})} = 0.31$ (TEO), 0.012 (TE), β_1 = -0.042, 0.25, LM (two-sided, same across figures); $p_{(\text{TEO-vs-TE})} > 0.05$ for day 1 to 8; for day 9, p = 0.036, permutation test (1000 permutations, two-sided, same across figures); X: $p_{(\text{effect-size-vs-day})}$ = 0.43, 0.67, β_1 = - 0.032, 0.035, LM; $p_{(\text{TEO-vs-TE})}$ = 0.17, 0.0033, 0.0025, 0.0064, 0.0025 (day 1-5), permutation test. $p_{(\text{region}\times\text{day})}$ = 0.008 (T), 0.46(X), LM. (**C**), same as (**A**), but for morph identity. T: $p_{(\text{percentage-vs-day})}$ = 0.95 (TEO), 0.065 (TE), r = - 0.025, 0.64; X: $p_{(\text{percentage-vs-day})}$ = 0.82, 0.25, r = - 0.15, 0.64, Pearson correlation. Lower bounds of one-tailed 95% confidence interval (CI) of $r_{(\text{TE-vs-day})} - r_{(\text{TEO-vs-day})}$: -0.31(T), -1.2 (X), bootstrapping Pearson correlation. (**D**), same as (**B**), but for morph identity. T: $p_{(\text{effect-size-vs-day})}$ = 0.83 (TEO), 0.036 (TE), β_1 = 0.069, 0.99, LM; $p_{(\text{TEO-vs-TE})} > 0.05$ for all days, permutation test; X: $p_{(\text{effect-size-vs-day})}$ = 0.77, 0.85, β_1 = 0.23, - 0.086, LM. $p_{(\text{TEO-vs-TE})}$ = 0.001 for all days, permutation test; $p_{(\text{region}\times\text{day})}$ = 0.099 (T), 0.72 (X), LM. permutation test. Data are mean ± SEM; p-values FDR-adjusted (Benjamini–Hochberg). *p < 0.05, **p < 0.01, ***p < 0.001.

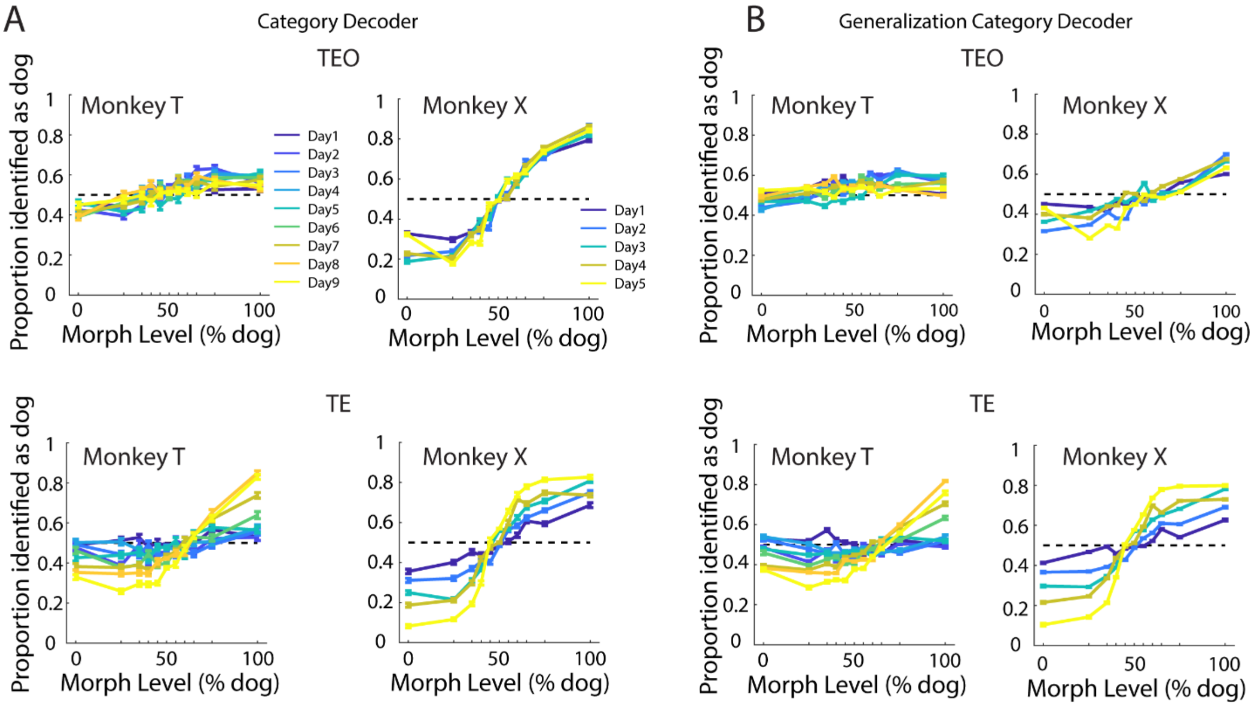

Supplementary Fig. 4. Categorization performance of decoders in each learning day. (A) Performance of traditional category decoder trained on TEO (top) and TE (bottom) neurons. (**B**) Performance of generalization category decoder trained on TEO (top) and TE (bottom) neurons. All data are represented as mean ± SEM.

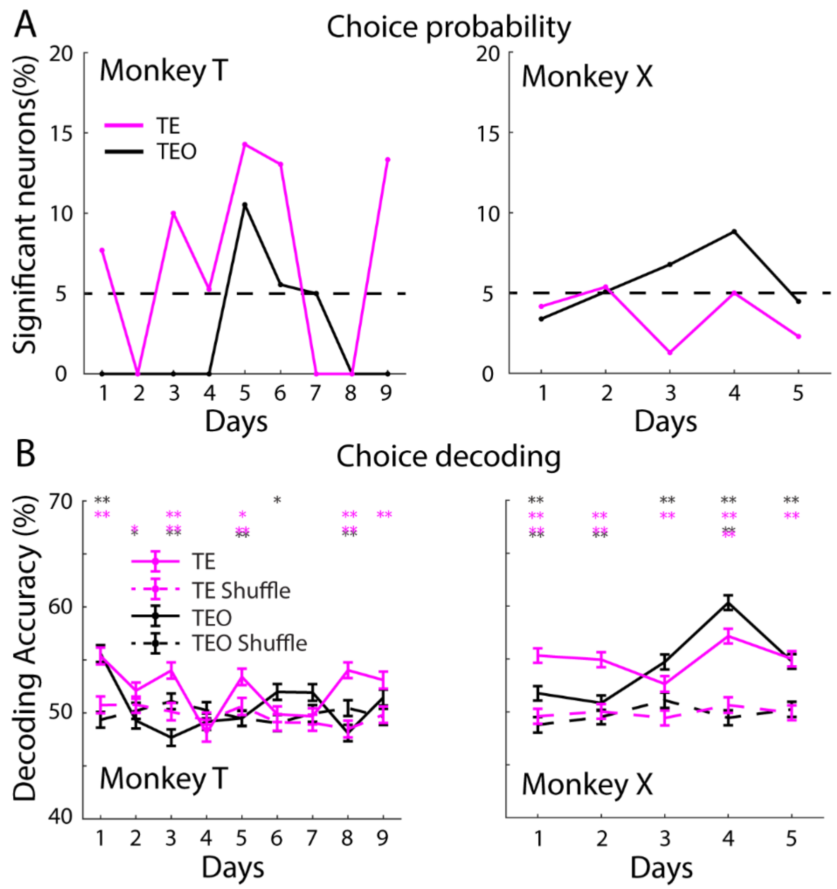

Supplementary Fig. 5. Choice-related signals in TE and TEO based on neural responses to 50% morph images. (A) Percentage of neurons with significant choice probability. For both monkeys and both regions, the percentages of significant neurons did not differ significantly from chance level (5%). $p > 0.05$ for all comparisons, Binomial test. **(B)** Decoding accuracy of choice decoder on population responses**.** T: $p_{(TE\text{--}vs\text{-}shuffle)}$ = 0.009, 0.43, 0.018, 0.43, 1, 0.033, 0.12, 0.083, 0.13 (day 1-9); $p_{(TE\text{-}vs\text{-}shuffle)}$ = 0.0045, 0.31, 0.006, 0.4, 0.032, 0.54, 0.59, 0.0045, 0.0067; $p_{(TEO\text{-}vs\text{-}TE)}$ = 0.86, 0.032, 0.0045, 0.41, 0.006, 0.067, 0.067, 0.0045, 0.17, permutation test; X: $p_{(TEO\text{-}vs\text{-}shuffle)}$ = 0.0025, 0.2, 0.0025, 0.0025, 0.0025 (day 1-5); $p_{(TE\text{-}vs\text{-}shuffle)}$ = 0.0012, 0.0012, 0.003, 0.0012, 0.0012; $p_{(TEO\text{-}vs\text{-}TE)}$ = 0.0025, 0.0025, 0.054, 0.0033, 0.083, permutation test. For both monkeys and both regions, choice decoding accuracies did not significantly increase with learning. T: 95% CI of $r_{(TEO)} - r_{(shuffle)}$: [-0.69 0.49], $r_{(TE)} - r_{(shuffle)}$: [-0.17 0.73]; X: 95% CI of $r_{(TEO)} - r_{(shuffle)}$: [-0.25 0.99], $r_{(TE)} - r_{(shuffle)}$: [-0.94 0.95], bootstrapping Pearson correlation, decoding accuracy vs day number. Data are mean ± SEM; p values were adjusted by Benjamini-Hochberg FDR correction. * $p < 0.05$. ** $p < 0.01$.

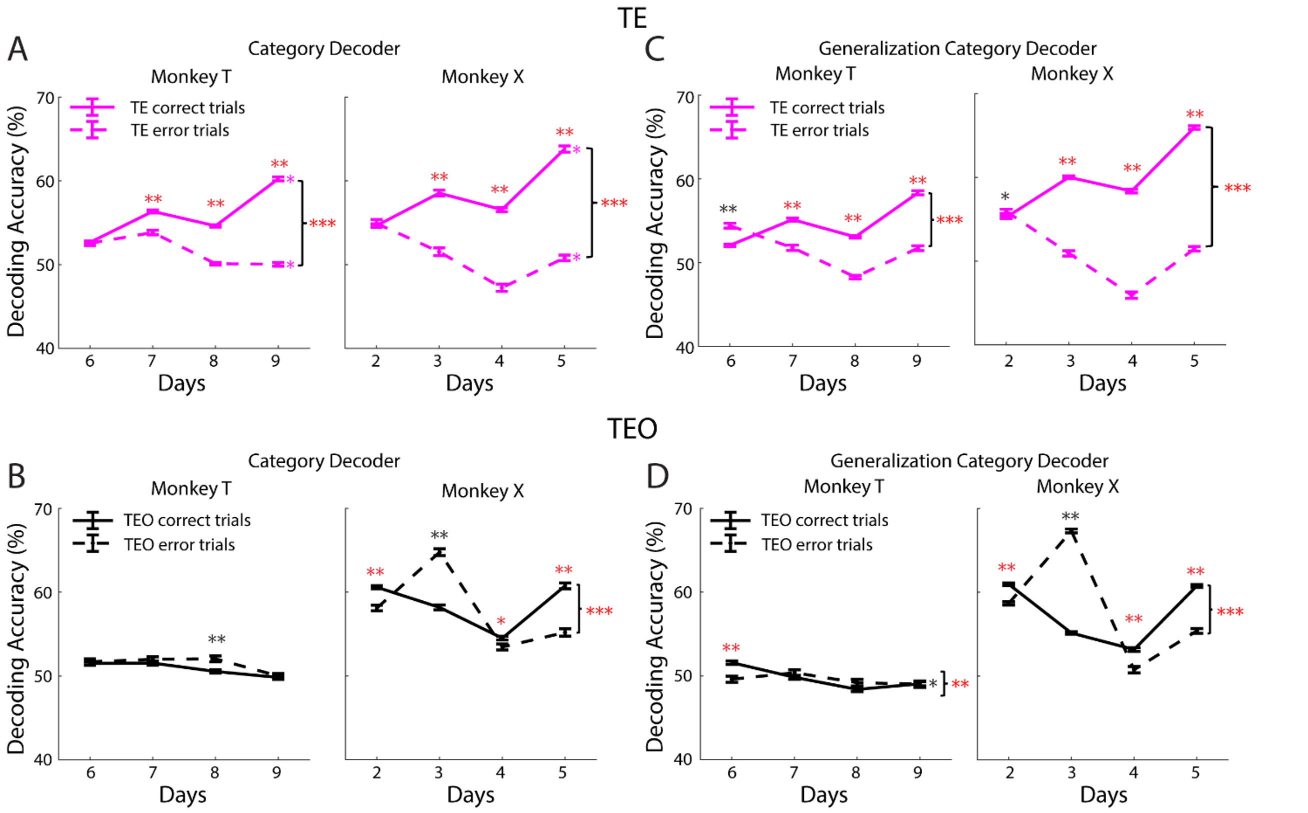

Supplementary Fig. 6. Decoding accuracy in correct and error trials. (**A**) Decoding accuracy of TE population in correct and error trials based on category decoder (Figure 3A). T: $p_{(correct\text{-}vs\text{-}error)}$ = 0.75, 0.0013, 0.0013, 0.0013 (day 6-9); X: $p_{(correct\text{-}vs\text{-}error)}$ = 0.56, 0.0013, 0.0013, 0.0013(day 2-5), permutation test. 95% CI of $r_{(TE\_correct)} - r_{(shuffle)}$: [0.02 1.82] (T), [0.12 1.76] (X); 95% CI of $r_{(TE\_error)} - r_{(shuffle)}$: [-1.8 -0.32], [-1.65 -0.06], bootstrapping Pearson correlation (5000 bootstrap samples two-sided, same across figures). $p_{(region\times day)} = 4.8 \times 10^{-93}$ (T), 1.6×10^{-59} (X), LM. Red and black asterisks atop the lines denote decoding accuracies for correct trials that are significantly higher and lower, respectively, than those for error trials. (**B**) same as (**A**), but for TEO. T: $p_{(correct\text{-}vs\text{-}error)}$ = 0.65, 0.49, 0.004, 0.65(day 6-9); X: $p_{(correct\text{-}vs\text{-}error)}$ = 0.0013, 0.0013, 0.016, 0.0013(day 2-5), permutation test. Either TEO decoding accuracy of correct or error trials significantly correlated with training days. 95% CI of $r_{(TEO\_correct)} - r_{(shuffle)}$: [-1.35 0.12] (T), [-0.69 0.88] (X); $r_{(TEO\_error)} - r_{(shuffle)}$: [-1.67 0.28], [-1.38 0.45], bootstrapping Pearson correlation. $p_{(region\times day)}$ = 0.57 (T), 3.6×10^{-11} (X), LM. (**C**) Decoding accuracy of TE population in correct and error trials based on generalization category decoder (Figure 3D). T: $p_{(correct\text{-}vs\text{-}error)}$ = 0.001 for all days; X: $p_{(correct\text{-}vs\text{-}error)}$ = 0.021, 0.0013, 0.0013, 0.0013(day 2-5), permutation test. Either TE decoding accuracy of correct or error trials significantly correlated with training days. 95% CI of $r_{(TE\_correct)} - r_{(shuffle)}$: [-0.06 1.75] (T), [-0.01 1.82] (X); 95% CI of $r_{(TE\_error)} - r_{(shuffle)}$: [-1.43 0.27], [-1.33 0.02], bootstrapping Pearson correlation. $p_{(region\times day)} = 9.1 \times 10^{-59}$ (T), 1.4×10^{-115} (X), LM. (**D**) same as (**C**), but for TEO population. T: $p_{(correct\text{-}vs\text{-}error)}$ = 0.004, 0.28, 0.15, 0.92(day

6-9); X: $p_{(\text{correct-vs-error})} = 0.001$ for all days, permutation test. 95% CI of $r_{(\text{TEO\_correct})} - r_{(\text{shuffle})}$: [-1.55 -0.18] (T), [-0.94 0.89] (X); $r_{(\text{TEO\_error})} - r_{(\text{shuffle})}$: [-1.25 0.74], [-0.43 0.49], bootstrapping Pearson correlation. $p_{(\text{region}\times\text{day})} = 0.003$ (T), 3.2×10^{-21} (X), LM. Data are mean ± SEM; *p*-values FDR-adjusted (Benjamini–Hochberg). $^*p < 0.05$, $^{**}p < 0.01$, $^{***}p < 0.001$.

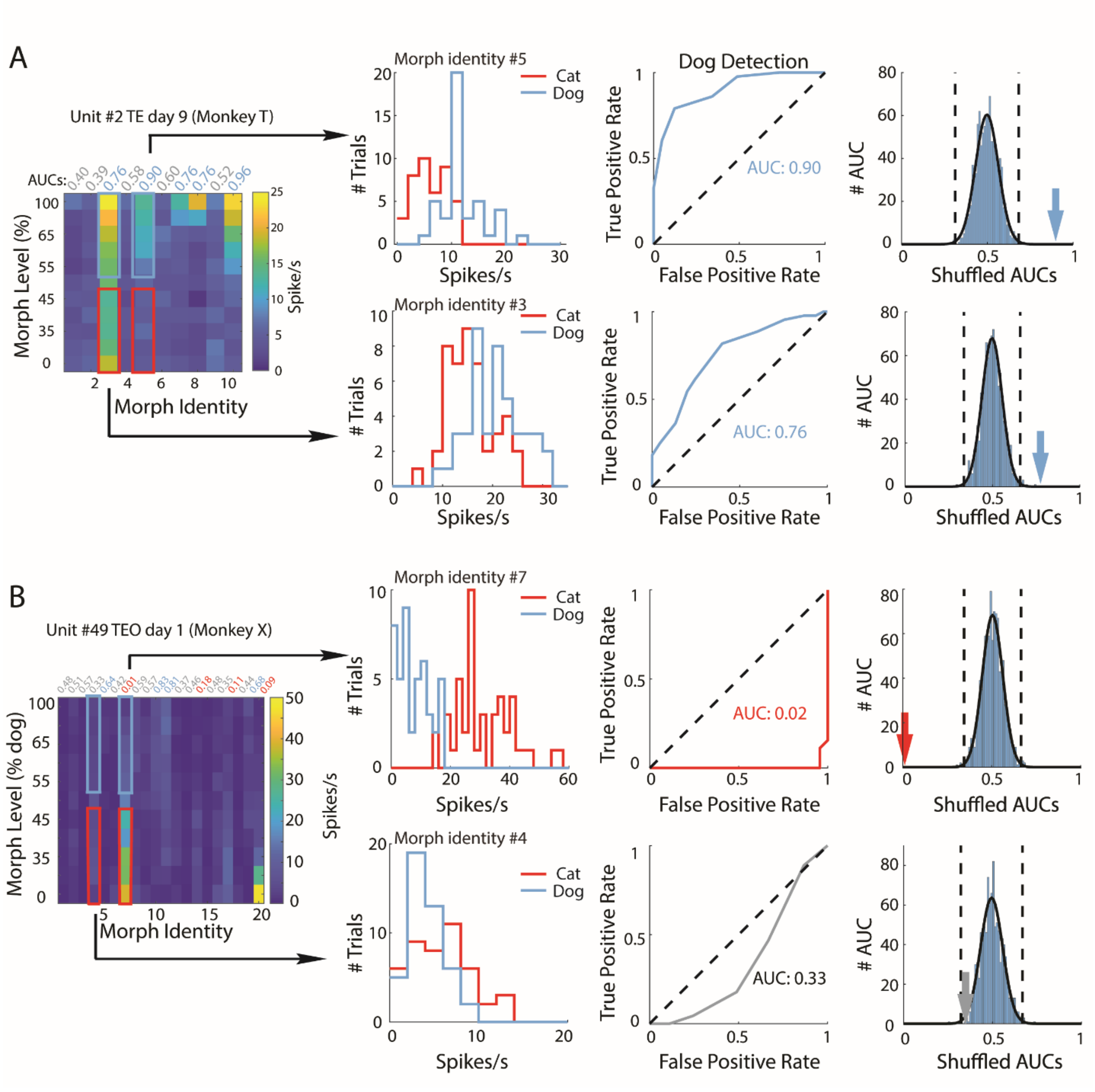

Supplementary Fig. 7. Example ROC analysis. Similar as Figure 6 (**A**) and (**B**). (**A**) A neuron from TE. (**B**) A neuron from TEO. Blue, red, and grey numbers on the top of color-coded neural responses indicated significant dog, cat, and nonsignificant AUCs, respectively.

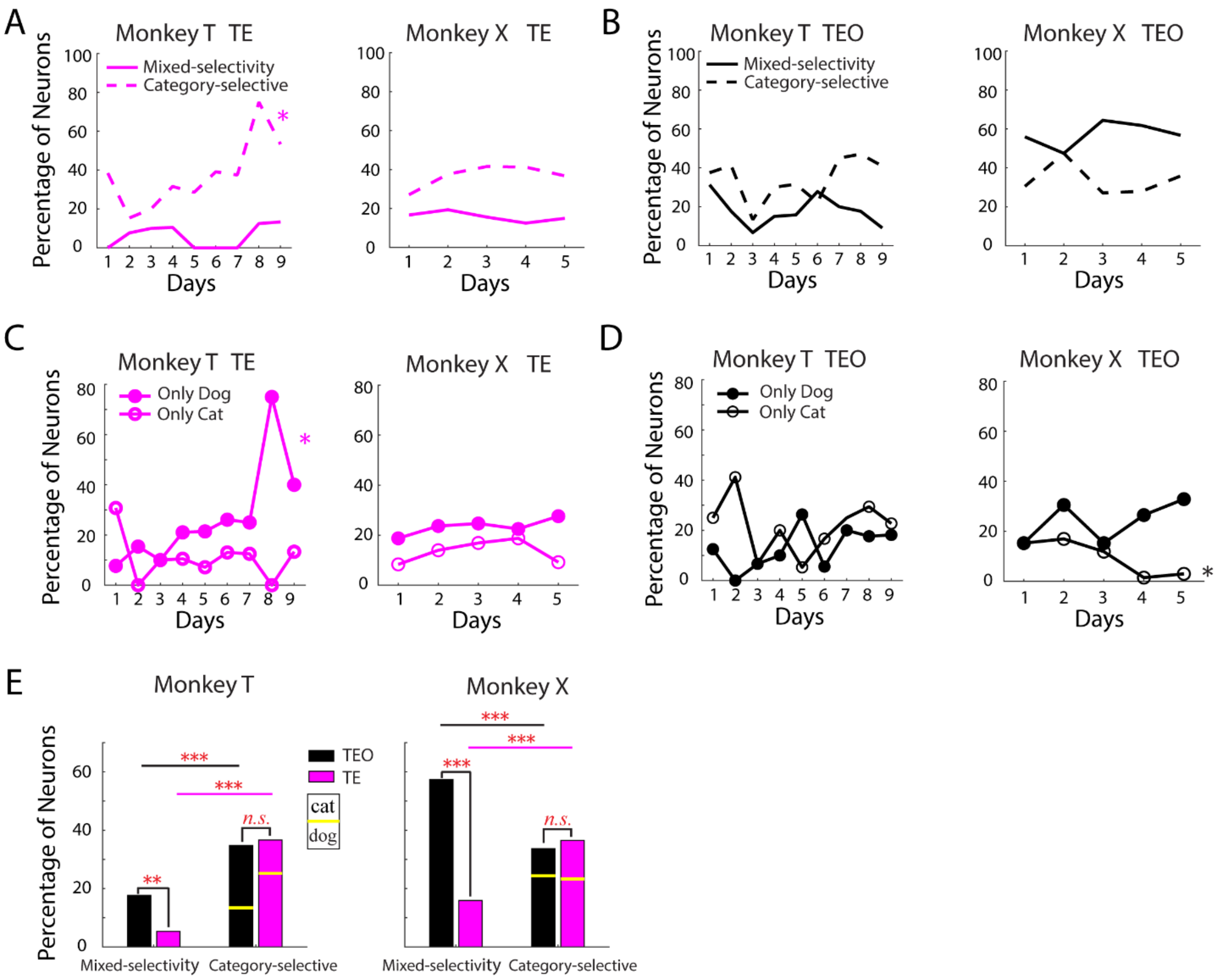

Supplementary Fig. 8. Coding properties from ROC analysis. (**A**) Percentage of mixed-selectivity and category-selective neurons in TE. T: $p_{(percentage\text{-}vs\text{-}day)} = 0.03$ (category-selective), 0.45 (mixed-selectivity), $r = 0.72$, 0.29; X: $p_{(percentage\text{-}vs\text{-}day)} = 0.26$, 0.23, $r = 0.62$, - 0.65, Pearson correlation. (**B**) same as (**A**), but for TEO. T: $p_{(percentage\text{-}vs\text{-}day)} = 0.35$ (category-selective), 0.46 (mixed-selectivity), $r = 0.36$, -0.28; X: $p_{(percentage\text{-}vs\text{-}day)} = 0.79$, 0.52, $r = -0.17$, 0.39, Pearson correlation. (**C**) Percentage of TE category-selective neurons preferring dogs and cats. T: $p_{(percentage\text{-}vs\text{-}day)} = 0.016$ (dog), 0.41(cat), $r = 0.77$, - 0.31; X: $p_{(percentage\text{-}vs\text{-}day)} = 0.097$, 0.72, r = 0.81, 0.22, Pearson correlation. (**D**) Same as (**C**), but for TEO. T: $p_{(percentage\text{-}vs\text{-}day)} = 0.13$ (dog), 0.91(cat), $r = 0.54$, -0.046; X: $p_{(percentage\text{-}vs\text{-}day)} = 0.3$, 0.041, $r = 0.59$, -0.89, Pearson correlation. (**E**), TE neurons showed stronger category preference than TEO neurons, pooled across days. mixed-selectivity VS category-selective: T: $p = 1.3 \times 10^{-9}$ (TE), 7×10^{-4} (TEO), $\chi^2_{(1)} = 36.8$, 11.5; X: $p = 1.05 \times 10^{-11}$, 4.4×10^{-9}, $\chi^2_{(1)} = 46.2$, 34.4. TE vs TEO: T: $p = 0.0024$ (mixed-selectivity), 0.83 (category-selective), $\chi^2_{(1)} = 9.23$, 0.046; $p = 2.2 \times 10^{-16}$, 0.47, $\chi^2_{(1)} = 138.34$, 0.52, chi-square test. Data are mean ± SEM; p-values FDR-adjusted (Benjamini–Hochberg). $^*p < 0.05$, $^{**}p < 0.01$, $^{***}p < 0.001$.

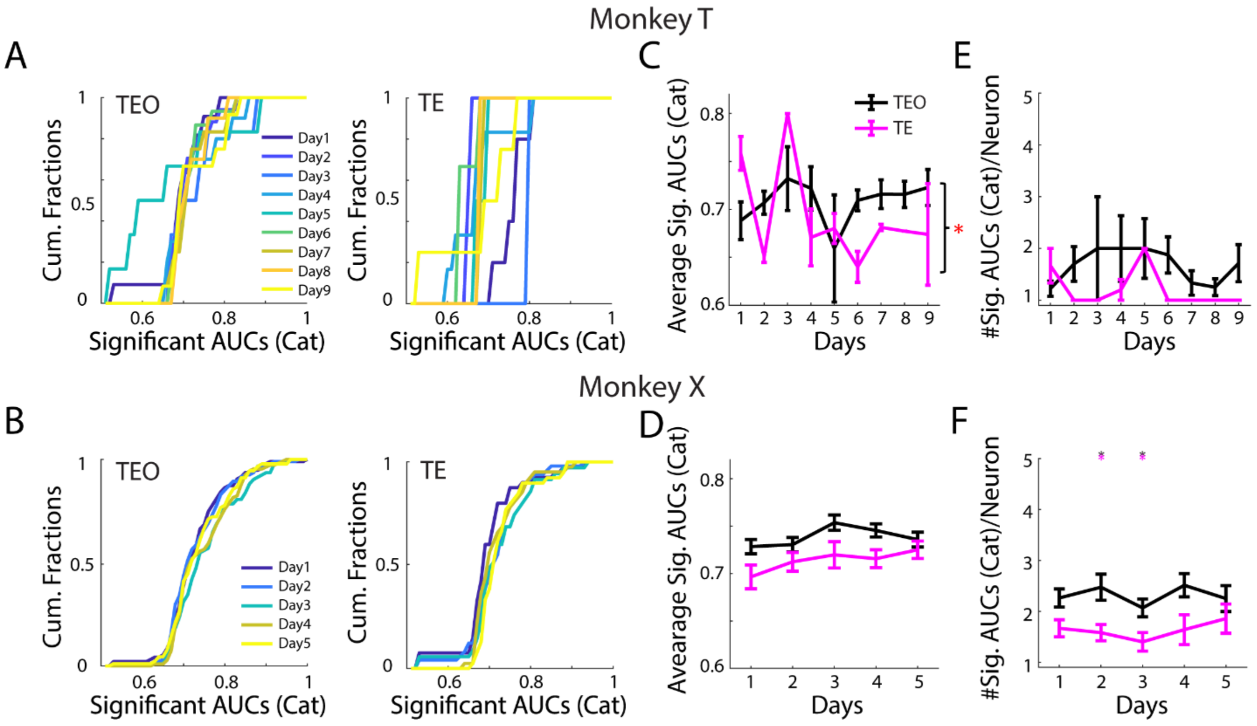

Supplementary Fig. 9. Encoding of cat images was not enhanced in either TE or TEO. (**A**) and (**B**), Cumulative distribution of significant cat AUCs. (**C**) and (**D**) Average significant cat AUCs. T: $p_{(\text{AUC-vs-day})} = 0.34$ (TEO), 0.07 (TE), $\beta_1 = 0.0023$, -0.009, LM. $p_{(\text{TEO-vs-TE})} > 0.05$ for all days, permutation test; n(TEO) = 11, 17, 6, 10, 6, 15, 12, 10, 12 significant cat AUCs (day 1-9); n(TE) = 5, 2, 1, 6, 2, 3, 2, 1, 4. X: $p_{(\text{AUC-vs-day})} = 0.2009$ (TEO), 0.088 (TE), $\beta_1 = 0.0031$, 0.0059, LM. $p_{(\text{TEO-vs-TE})} > 0.05$ for all days, permutation test. n(TEO) = 95, 94, 93, 108, 90 significant cat AUCs (day 1-5); n(TE) = 40, 49, 35, 41, 39. $p_{(\text{region}\times\text{day})} = 0.049$ (T), 0.74 (X), LM. (**E**) and (**F**) Number of significant cat AUCs in neurons with ≥1 significant cat AUC. T: $p_{(\#\text{AUC-vs-day})} = 0.94$ (TEO), 0.06 (TE), $\beta_1 = -0.003$, -0.06, LM. $p_{(\text{TE-vs-TEO})} > 0.05$ for all days, permutation test. n(TEO) = 9, 10, 3, 5, 3, 8, 9, 8, 7 neurons (day 1-9); n(TE) = 3, 2, 1, 5, 1, 3, 2, 1, 4; X: $p_{(\#\text{AUC-vs-day})} = 0.95$, 0.57, $\beta_1 = 0.004$, 0.04, LM. $p_{(\text{TEO-vs-TE})} = 0.057$, 0.025, 0.038, 0.39, 0.41 (day 1-5), permutation test. n(TEO) = 42, 38, 45, 43, 40 neurons (day 1-5); n(TE) = 24, 31, 25, 25, 21. $p_{(\text{region}\times\text{day})} = 0.42$ (T), 0.74 (X), LM. Data are mean ± SEM; p-values FDR-adjusted (Benjamini–Hochberg). $^*p < 0.05$, $^{**}p < 0.01$, $^{***}p < 0.001$.

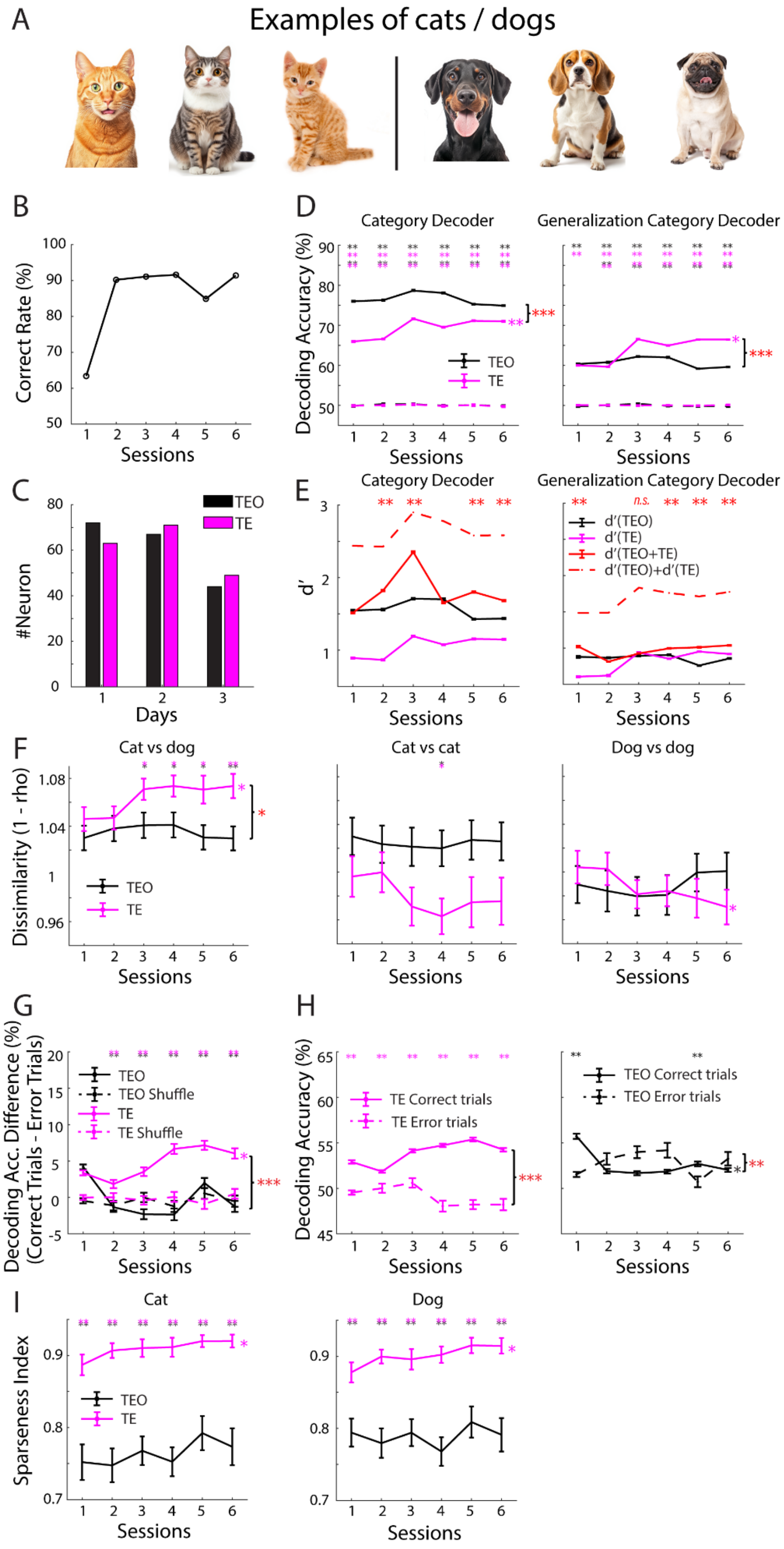
A
Examples of cats / dogs
B
Correct Rate (%)
Sessions
C
#Neuron
Days
TEO
TE
D
Category Decoder
Generalization Category Decoder
Decoding Accuracy (%)
Sessions
E
d'
d'(TEO)
d'(TE)
d'(TEO+TE)
d'(TEO)+d'(TE)
n.s.
F
Cat vs dog
Cat vs cat
Dog vs dog
Dissimilarity (1 - rho)
G
Decoding Acc. Difference (%)
(Correct Trials - Error Trials)
TEO Shuffle
TE Shuffle
H
TE Correct trials
TE Error trials
TEO Correct trials
TEO Error trials
I
Cat
Dog
Sparseness Index

Supplementary Fig. 10. Neural plasticity during learning of 20-cat/20-dog task. (**A**) Example images. Images designed by Freepik (www.freepik.com). (**B**) Monkey's performance. (**C**) Neuron counts. (**D**) Decoding accuracy. Left: 95% CI of $r_{(TEO)}$ - $r_{(shuffle)}$: [-0.83 0.52], 99% CI of $r_{(TE)}$ - $r_{(shuffle)}$: [0.12 1.71], bootstrapping Pearson correlation; $p_{(region \times day)} = 1.2 \times 10^{-135}$, LM; $p_{(TEO\text{-}vs\text{-}TE)} = 0.001$, $p_{(TEO\text{-}vs\text{-}shuffle)} = 0.001$, $p_{(TE\text{-}vs\text{-}shuffle)} = 0.001$ for all sessions, permutation test; Right: 95% CI of $r_{(TEO)}$ - $r_{(shuffle)}$: [-0.77 0.35], 95% CI of $r_{(TE)}$ - $r_{(shuffle)}$: [0.11 1.58]; $p_{(region \times day)} = 2.8 \times 10^{-297}$; $p_{(TEO\text{-}vs\text{-}TE)}$ = 0.066,0.0012, 0.0012, 0.0012, 0.0012, 0.0012 (session 1-6), $p_{(TEO\text{-}vs\text{-}TE)} = 0.001$, $p_{(TEO\text{-}vs\text{-}shuffle)} = 0.001$, $p_{(TE\text{-}vs\text{-}shuffle)} = 0.001$ for all sessions. (**E**) Category discriminability. Left: p = 0.019, 0.0015, 0.0015, 0.0048, 0.0015, 0.0015 (session 1-6); Right: p = 0.0012, 0.0012, 0.089, 0.0012, 0.0012, 0.0012, permutation test. (**F**) Representational dissimilarity. $p_{(TE\text{-}vs\text{-}session)} = 0.011$ (cat-vs-dog), 0.21 (cat-vs-cat), 0.041 (dog-vs-dog); $p_{(TEO\text{-}vs\text{-}session)}$ = 0.78, 0.93, 0.44, LM; $p_{(region \times session)}$ = 0.049, 0.38, 0.056, LM. Left: $p_{(TEO\text{-}vs\text{-}TE)}$ = 0.29, 0.53, 0.051, 0.046, 0.039, 0.006 (session 1-6); middle: $p_{(TEO\text{-}vs\text{-}TE)}$ = 0.18, 0.31, 0.076, 0.03, 0.076, 0.076; right: $p_{(TEO\text{-}vs\text{-}TE)}$ = 0.74, 0.74, 0.93, 0.93, 0.74, 0.74, permutation test. (**G**) Decoding accuracy difference (correct vs. error). $p_{(TE\text{-}vs\text{-}TEO)}$ = 0.091, 0.0024, 0.0015, 0.0015, 0.0015, 0.0015 (session 1-6), permutation test. 95% CI of $r_{(TE)}$ - $r_{(shuffle)}$: [0.1 1.47]; 95% CI of $r_{(TEO)}$ - $r_{(shuffle)}$: [-1.12 0.16], bootstrapping Pearson correlation. $p_{(region \times session)} = 6.3 \times 10^{-11}$, LM. (**H**) Decoding accuracy. TE: $p_{(correct\ vs\ error)}$ = 0.0012, 0.002, 0.0012, 0.0012, 0.0012, 0.0012 (session 1-6), permutation test. 95% CI of $r_{(correct)}$ - $r_{(shuffle)}$: [-0.02 1.42]; 95% CI of $r_{(error)}$ - $r_{(shuffle)}$: [-1.41 0.07], bootstrapping Pearson correlation. $p_{(trial\_type \times session)} = 2.2 \times 10^{-13}$, LM. TEO: $p_{(correct\text{-}vs\text{-}error)}$ = 0.003, 0.067, 0.003, 0.009, 0.004, 0.11(session 1-6). 99% CI of $r_{(correct)}$ - $r_{(shuffle)}$: [-1.59 -0.36]; 95% CI of $r_{(error)}$ - $r_{(shuffle)}$: [-1.42 0.06]. $p_{(trial\_type \times session)} = 0.003$. (**I**) Sparseness of responses. Cat: $p_{(TEO\text{-}vs\text{-}TE)} = 0.001$ for all sessions, permutation test. $p_{(sparseness\text{-}vs\text{-}session)} = 0.29$ (TEO), 0.046 (TE), LM; $p_{(region \times session)} = 0.98$, LM. Dog: $p_{(TEO\text{-}vs\text{-}TE)}$ = 0.002, 0.0012, 0.0012, 0.0012, 0.0012, 0.0012 (session 1-6). $p_{(sparseness\text{-}vs\text{-}session)} = 0.9$ (TEO), 0.027 (TE); $p_{(region \times session)}$ = 0.3.

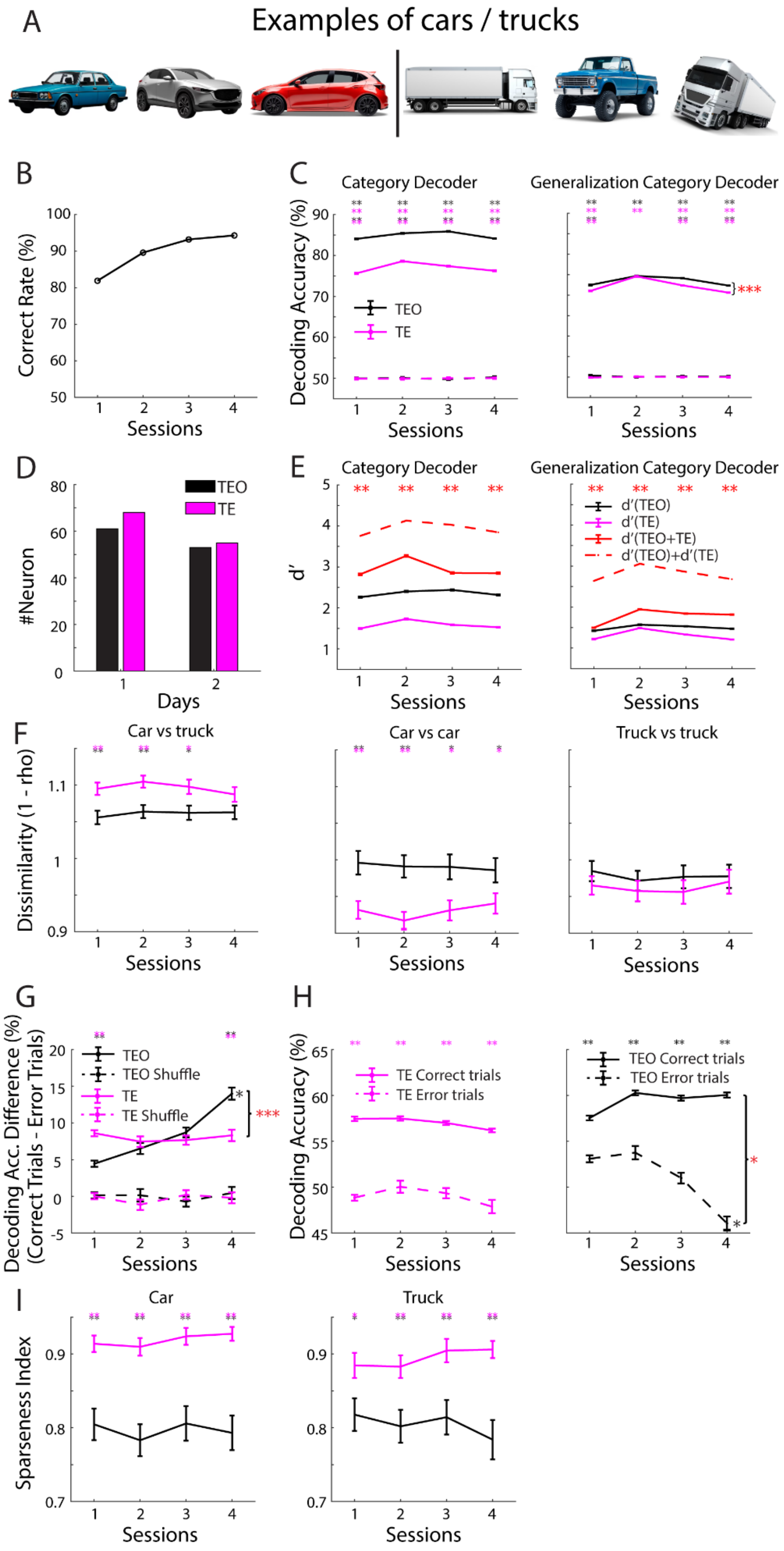
A
Examples of cars / trucks
B
Correct Rate (%)
Sessions
C
Category Decoder
Generalization Category Decoder
Decoding Accuracy (%)
TEO
TE
Sessions
D
TEO
TE
#Neuron
Days
E
Category Decoder
Generalization Category Decoder
d'
d'(TEO)
d'(TE)
d'(TEO+TE)
d'(TEO)+d'(TE)
Sessions
F
Car vs truck
Car vs car
Truck vs truck
Dissimilarity (1 - rho)
Sessions
G
Decoding Acc. Difference (%)
(Correct Trials - Error Trials)
TEO
TEO Shuffle
TE
TE Shuffle
Sessions
H
Decoding Accuracy (%)
TE Correct trials
TE Error trials
TEO Correct trials
TEO Error trials
Sessions
I
Car
Truck
Sparseness Index
Sessions

Supplementary Fig. 11. Neural plasticity during learning of 20-car/20-truck task. (**A**) Example images. Images designed by Freepik (www.freepik.com). (**B**) Monkey's performance. (**C**) Neuron counts. (**D**) Decoding accuracy. Left: 95% CI of $r_{(TEO)} - r_{(shuffle)}$: [-1.18 0.48], 95% CI of $r_{(TE)} - r_{(shuffle)}$: [-0.34 1.07], bootstrapping Pearson correlation; $p_{(region \times session)} = 0.96$, LM; $p_{(TE\text{-}vs\text{-}shuffle)} = 0.001$, $p_{(TEO\text{-}vs\text{-}shuffle)} = 0.001$, $p_{(TEO\text{-}vs\text{-}TE)} = 0.001$ for all sessions, permutation test; Right: 95% CI of $r_{(TEO)} - r_{(shuffle)}$: [-1.2 0.6], 95% CI of $r_{(TE)} - r_{(shuffle)}$: [-0.83 0.87]; $p_{(region \times session)} = 0.0002$; $p_{(TE\text{-}vs\text{-}shuffle)} = 0.001$, $p_{(TEO\text{-}vs\text{-}shuffle)} = 0.001$ for all sessions; $p_{(TEO\text{-}vs\text{-}TE)} = 0.0013$, 0.57, 0.0013, 0.0013 (session 1-4). (**E**) Category discriminability. $p = 0.001$ for comparisons, permutation test. (**F**) Representational dissimilarity. $p_{(TEO\text{-}vs\text{-}session)} = 0.64$ (cat-vs-dog), 0.67(cat-vs-dog), 0.8(dog-vs-dog); $p_{(TE\text{-}vs\text{-}session)} = 0.46$, 0.4, 0.83; $p_{(region \times session)} = 0.74$, 0.4, 0.44, LM. Left: $p_{(TE\text{-}vs\text{-}TEO)} = 0.004$, 0.004, 0.019, 0.07 (session 1-4); middle: $p_{(TE\text{-}vs\text{-}TEO)} = 0.008$, 0.004, 0.013, 0.035; right: $p_{(TE\text{-}vs\text{-}TEO)} = 0.63$, 0.63, 0.63, 0.74, permutation test. (**G**) Decoding accuracy difference (correct vs. error). $p_{(TEO\text{-}vs\text{-}TE)} = 0.002$, 0.35, 0.34, 0.002 (session 1-4), permutation test. 95% CI of $r_{(TE)} - r_{(shuffle)}$: [-1.43 1.03]; 95% CI of $r_{(TEO)} - r_{(shuffle)}$: [0.1 1.86], bootstrapping Pearson correlation test. $p_{(region \times session)} = 8.5 \times 10^{-14}$, LM. (**H**) Decoding accuracy in correct and error trials. TE: $p_{(correct\text{-}vs\text{-}error)} = 0.001$ for all sessions, permutation test. 95% CI of $r_{(correct)} - r_{(shuffle)}$: [-1.84 0.12]; 95% CI of $r_{(error)} - r_{(shuffle)}$: [-1.36 0.7], bootstrapping Pearson correlation. $p_{(trial\_type \times session)} = 0.8$, LM. TEO: $p_{(correct\text{-}vs\text{-}error)} = 0.001$ for all sessions, permutation test. 95% CI of $r_{(correct)} - r_{(shuffle)}$: [-0.13 1.72]; 95% CI of $r_{(error)} - r_{(shuffle)}$: [-1.74 -0.01], bootstrapping Pearson correlation. $p_{(trial\_type \times session)} = 1.2 \times 10^{-23}$, LM. (**I**) Sparseness index. Car: $p_{(TEO\text{-}vs\text{-}TE)} = 0.001$ for all sessions, permutation test. $p_{(sparseness\text{-}vs\text{-}session)} = 0.9$ (TE), 0.29 (TEO), LM; $p_{region \times session)} = 0.54$, LM. Truck: $p_{(TEO\text{-}vs\text{-}TE)} = 0.017$, 0.004, 0.004, 0.004 (session 1-4), permutation test. $p_{(sparseness\text{-}vs\text{-}session)} = 0.4$ (TEO), 0.21(TE), LM; $p_{(region \times session)} = 0.16$, LM. Data are mean ± SEM; p-values FDR-adjusted (Benjamini–Hochberg). *$p < 0.05$, **$p < 0.01$, ***$p < 0.001$.

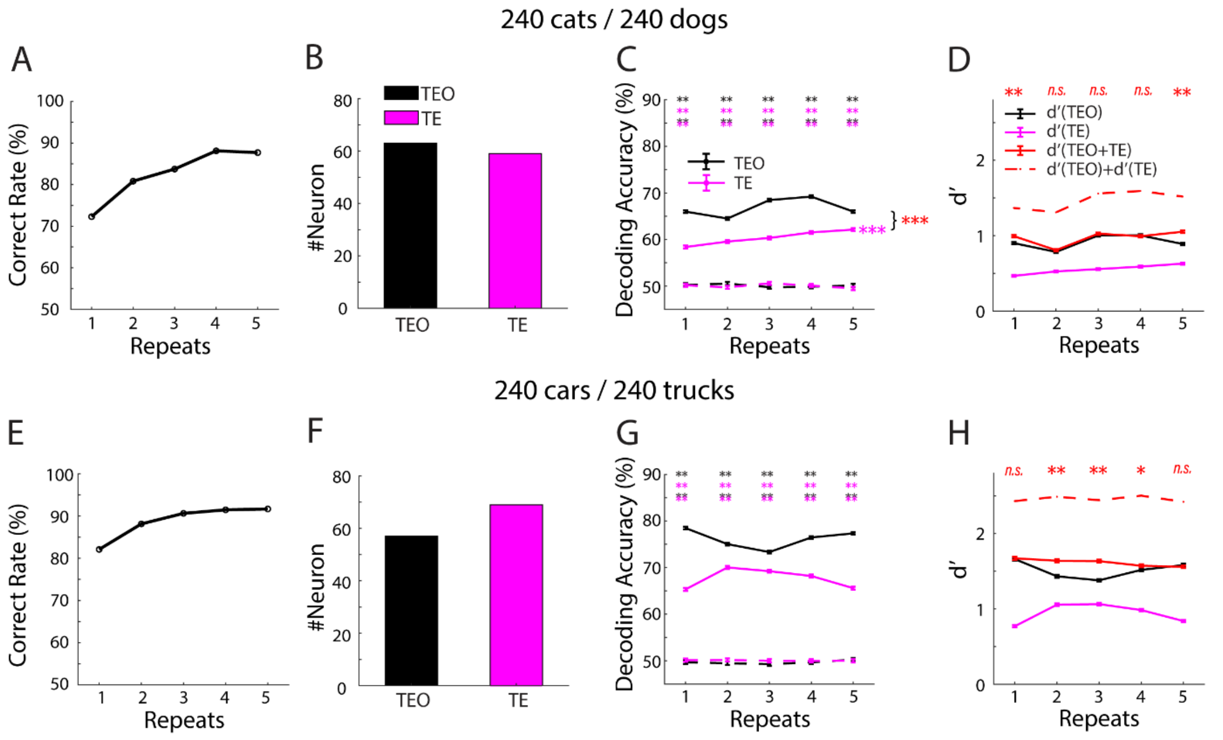

Supplementary Fig. 12. Neural plasticity during learning of non-morphed 240-cat/240-dog and 240-car/240-truck categorization tasks. (**A**) Monkey X's performance on each repeat of 240 cats and 240 dogs within one single day. (**B**) Number of single units recorded from TE and TEO. (**C**) Category decoding accuracy for cats and dogs of TEO (black) and TE (magenta) neuron populations. Decoding accuracies of TE, not TEO, significantly increased across repeats. 95% CI of $r_{(TEO)}$ - $r_{(shuffle)}$: [-0.2 1.31], 99% CI of $r_{(TE)}$ - $r_{(shuffle)}$: [0.06 1.95], bootstrapping Pearson correlation test, decoding accuracy vs repeat number. For both TE and TEO, decoding accuracies were significantly stronger than shuffled data on each repeat, $p = 0.001$ for all repeats, permutation test. Decoding accuracies of TEO were significantly stronger than TE on all repeats, $p = 0.001$ for all repeats, permutation test. Interaction between brain regions and repeats was significant ($p = 0.0006$, LM). (**D**) Category discriminability. Notations are the same as Figure 3C, F. p = 0.0025, 0.34, 0.34, 0.6, 0.0025 (repeat 1-5), permutation test. (**E**) and (**F**) same as (**A**) and (**B**), but for 240 cars and 240 trucks. (**G**) same as (**C**), but for cars and trucks. Decoding accuracies of either TE or TEO significantly increased across repeats. 95% CI of $r_{(TEO)}$ - $r_{(shuffle)}$: [-1 0.36], 95% CI of $r_{(TE)}$ - $r_{(shuffle)}$: [-0.89 0.85], bootstrapping Pearson correlation, decoding accuracy vs repeat number. For both TE and TEO, decoding accuracies were significantly stronger than shuffled data on each repeat, $p = 0.001$ for all repeat, permutation test. Decoding

accuracies of TEO were significantly stronger than TE on all repeats, $p = 0.001$ for all repeats, permutation test. Interaction between brain regions and repeats was not significant ($p = 0.74$, LM). (**H**) same as (**D**), but for cars and trucks. $p = 0.7, 0.0025, 0.0025, 0.045, 0.48$ (repeat 1-5), permutation test. Data are mean ± SEM; *p*-values FDR-adjusted (Benjamini–Hochberg). *$p < 0.05$, **$p < 0.01$, ***$p < 0.001$.

Notes for categorization task with 20-cat/20-dog, 240-cat/240-dog, 20-car/20-truck and 240-car/ 240-truck

For monkey X, the tasks were administered in the following order: (1) 20-cat vs. 20-dog categorization, (2) 240-cat vs. 240-dog categorization, (3) morphed cat–dog categorization, (4) 20-car vs. 20-truck categorization, and (5) 240-car vs. 240-truck categorization.

In the 20-cat/20-dog task (3 days) and the 20-car/20-truck task (2 days), each day's trials were split into two equal sessions, with 25 repetitions of every image per session. For the 240-cat/240-dog and 240-car/240-truck tasks, neural activity was recorded in a single day. Trials were divided into five sequential segments, each containing one presentation of every image.

Analyses were conducted on responses from the 0–530 ms window, identical to the window used for the morphed-image task. The category decoder was identical to the one used for the morphed cat–dog analyses. For each image, trials were randomly split into training (~80 %), validation (~10 %), and test (~10 %) sets. For each session, decoding was run 500 times over different random selections of neurons and trials. The number of randomly selected neurons in each repeat fixed to the smallest population recorded across all days and brain regions to maintain comparability.

For generalization category decoding, 10-fold cross-validation at the image level was performed. For trials of training images, ~10% was used for validation, the remaining trials were used for decoder training. All the trials of testing images were used for test. Cross-validation was repeated for 500 times over different random sampling of neurons and trials.

For the 240-cat/240-dog and 240-car/240-truck datasets, each image appeared only once per session. Therefore, we applied generalization decoding only. Images were randomly split into training (~80 %), validation (~10 %), and test (~10 %) sets. The decoding was repeated for 500 times.

For decoding on correct and error trials of 20-cat/20-dog and 20-car/20-truck tasks, only generalization decoding was performed due to the distribution and limitation of error trials. The monkey's high performance yielded very few error trials, and some images produced almost only errors, leaving too few correct trials for training. These images can only be used for testing, which was consistent with the paradigm of generalization decoder. Thus, traditional category

decoding (Figure 3A) was not feasible and only the generalization decoding was performed (Figure 3D). To collect more error trials for testing, we adopted a 2-fold cross-validation strategy, instead of 10-fold. Cross-validation was repeated for 500 times over different random sampling of neurons and trials.

Single-unit sparseness index (Figure S9I, S10I) was calculated as following:

$$S = \frac{(\sum_{i=1}^{n} \frac{R_i}{n})^2}{\sum_{i=1}^{n} (\frac{R_i^2}{n})}$$

R_i represents the trial-averaged response to stimulus i. Sparseness index was calculated separately for cats and dogs. Higher sparseness index indicates more even distributed responses over stimuli. ROC-based analysis was not performed because there was no pairs of cat and dog as in the morphed cat/dog categorization task.

For 240 cats / 240 dogs, we detected a modest but significant same day rise in TE population decoding accuracy. No significant changes emerged in representational dissimilarity, the decoding difference between correct and error trials, or single-unit sparseness in either TE or TEO. These findings imply that larger-scale representational changes may happen over a longer timescale—potentially requiring overnight consolidation.
